# Training the salmon’s genes: influence of aerobic exercise, swimming performance and selection on gene expression in Atlantic salmon

**DOI:** 10.1186/s12864-017-4361-7

**Published:** 2017-12-15

**Authors:** Nicholas A. Robinson, Gerrit Timmerhaus, Matthew Baranski, Øivind Andersen, Harald Takle, Aleksei Krasnov

**Affiliations:** 1 Nofima, Osloveien 1, 1430 Ås, Norway; 2 Sustainable Aquaculture Laboratory - Temperate and Tropical (SALTT), School of BioSciences, The University of Melbourne, Parkville, Vic 3010 Australia; 3 Nofima, PO Box 210, 1431 Ås, Norway

**Keywords:** *Salmo salar*, Exercise, Swimming performance, Gene expression, SNP polymorphisms, Immune function, Natural selection, Selective breeding

## Abstract

**Background:**

Farmed and wild Atlantic salmon are exposed to many infectious and non-infectious challenges that can cause mortality when they enter the sea. Exercise before transfer promotes growth, health and survival in the sea. Swimming performance in juveniles at the freshwater parr stage is positively associated with resistance to some diseases. Genetic variation is likely to affect response to exercise. In this study we map genetic differences associated with aerobic exercise, swimming performance and genetic origin.

Eggs from the selectively bred Bolaks salmon and wild Lærdal River salmon strains were reared until parr in a common environment. Swimming performance was assessed by subjecting the fish to either continuous hard exercise or control conditions for 18 days. Heart was sampled for examination of gene expression using RNA-seq (~60 fish/treatment).

**Results:**

Lower expression of genes affecting immune function was found in domesticated than wild parr. Among wild parr under control exercise the expression of a large number of genes involved in general metabolism, stress and immune response was lower in superior swimmers suggesting that minimisation of energy expenditure during periods of low activity makes parr better able to sustain bursts of swimming for predator avoidance. A similar set of genes were down-regulated with training among wild parr with inferior swimming performance. These parr react to training in a way that their cardiac expression patterns become like the superior performing wild parr under control exercise conditions. Diversifying selection caused by breeding of domesticated stock, and adaptive pressures in wild stock, has affected the expression and frequency of single nucleotide polymorphisms (SNPs) for multiple functional groups of genes affecting diverse processes. SNPs associated with swimming performance in wild parr map to genes involved in energetic processes, coding for contractile filaments in the muscle and controlling cell proliferation.

**Conclusions:**

Domesticated parr have less phenotypic plasticity in response to training and lower expression of genes with functions affecting immune response. The genetic response to training is complex and depends on the background of parr and their swimming ability. Exercise should be tailored to the genetics and swimming performance of fish.

**Electronic supplementary material:**

The online version of this article (doi: 10.1186/s12864-017-4361-7) contains supplementary material, which is available to authorized users.

## Background

Fish health is one of the most important factors affecting aquaculture sustainability around the world. Over 1.2 million tonnes (NOK 44 billion value) of Atlantic salmon were farmed in Norway in 2014, and more than 10% of the 375 million animals farmed in the sea died from infectious diseases and other causes (Statistics Norway, www.ssb.no). When Atlantic salmon smolt are transferred from freshwater to the sea for aquaculture they are exposed to several infectious and non-infectious stressors that can cause mortality [1], and consequently, the majority of Atlantic salmon losses occur during the first months following sea transfer. Non-infectious causes of mortality for salmon in aquaculture include cardiac failure resulting from atherosclerosis, hypoplasia and other cardiac malformations and defects [2–10], much the same as those observed with “lifestyle diseases” in humans. Physical activity is an important factor affecting cardiovascular health in man [11], and is also likely to be critically important for the fitness of salmon in both the wild and aquaculture environments.

Although cardiovascular problems are typically detected among adult salmon in the sea phase of aquaculture production, there is increasing evidence that the course of cardiovascular fitness is set with the production routines used in earlier freshwater phases. Wild smolt have an angular pyramid shaped heart, whereas the heart of domesticated salmon is more rounded and consequently has reduced output and function compared with the heart of wild salmon [5, 10]. A high proportion of smolt from domesticated stock have high levels of fat deposition in the ventricle (44% compared to 0% in wild smolt) [10]. Exercise of salmon before transfer has been shown to be important for promoting growth, health and survival in the sea with aerobic exercise and swimming performance in Atlantic salmon associated with better protection against bacterial and viral diseases [12, 13]. Training juvenile salmon could therefore result in improved welfare, efficiency and profitability for farming in the sea.

Genetic changes resulting from natural selection, selective breeding, genetic drift and inbreeding are likely to influence how the stock responds to and benefits from exercise. Salmonid smolt migrating from fresh to salt water encounter many dangerous predators [14–19] and intense natural selection for swimming performance and fitness is therefore likely in many wild salmon populations. Evidence suggests that individuals that are more vulnerable to physiological stress with the transition from fresh to seawater may be subjected to greater predation as a consequence of less effective antipredator behaviours [20]. The vulnerable parr must expend large amounts of energy on frequent bursts of swimming activity as they migrate out to the open sea. Parr from longer fjords have been found to initiate migration earlier, move faster and are thought to be under greater selection pressure than parr from shorter fjords [21].

The Norwegian stock of Atlantic salmon used for aquaculture have been selectively bred since the 1970’s [22], with the first generations focussing on growth rate, and subsequent generations of breeding goals in the 1990’s adding some emphasis on resistance to specific diseases and specific quality traits. A number of different river stock were used as sources for establishing the selectively bred population of salmon [23]. Strong selection for specific traits, combined with genetic drift and absence of natural selective pressures for other traits (that increase the chance of survival in the wild), are likely to have affected the phenotype of the selectively bred population and contributed to the large phenotypic divergence between wild and domesticated fish.

Here we use RNA-seq to map genetic changes and differences in cardiac gene expression patterns associated with aerobic exercise, swimming performance and genetic origin with the ultimate aim of developing indicators in pre-smolt that could improve our understanding of key genes and mechanisms leading to greater robustness.

## Methods

### Experimental fish population

**Fig. 1:**
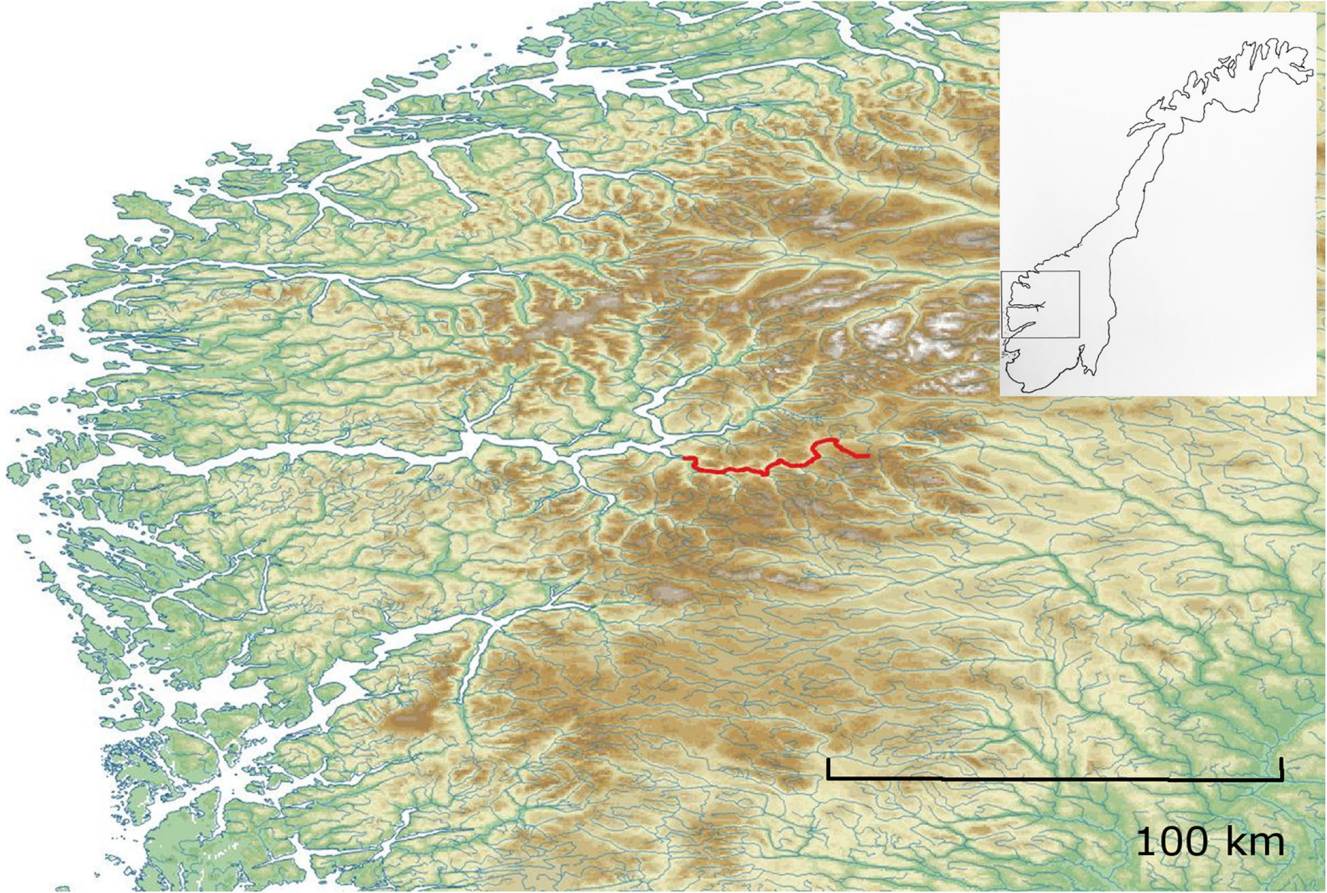
Source of wild parents for parr used in the study showing the main tributary of the Lærdal River (red) in Norway (inset). The figure was made using altitude raster data (30s resolution) obtained from the WorldClim dataset [78] and the open-source program QGIS v1.8

Two “strains” of Atlantic salmon were chosen for comparison. The Lærdal and Bolaks strains are characteristic Norwegian wild and domesticated Atlantic salmon, respectively. The Lærdal strain, from the Norwegian Lærdal River (61°N), was selected because of its relatively large spawning population size, genetic purity, genetic stability and a limited genetic influence from aquaculture escapees [24–27]. Its freshwater habitat has a large water discharge and low water temperature, which are typical conditions for wild Atlantic salmon in Norway [28, 29]. The Lærdal strain consists mainly of 2 and 3 sea-winter spawners and the migration route passes through the longest (>200 km) Norwegian fjord system, Sognefjorden (Fig. 1). The domesticated Bolaks strain originates from Western Norway, and the two founding stocks (for this strain) were from the Vosso (60°N) and Årøy (61°N) Rivers. For the first five generations until 2000, family-based selective breeding of the Bolaks strain focused on promoting rapid growth and delayed sexual maturation, after which specific disease resistance, fillet quality and coloration were also included as traits for selection.

The Bolaks experimental population was generated from eggs of seven females fertilized by two males and incubated at 7 °C by SalmoBreed (Bergen, Norway) until transportation to the Nofima research station, Sunndalsøra, Norway at 396 degree days (dd). The Lærdal experimental population was generated from eggs of five females that were fertilized by two males, and incubated at 7 °C in the local hatchery until transportation to the Nofima research station, Sunndalsøra, Norway at 410 dd. Eyed eggs of both populations were incubated in 5–6 °C freshwater until hatching, using side-by-side incubators (463–487 dd for Bolaks and 513–518 dd for Lærdal). Emergent fry were similarly reared under identical standard conditions and fed the same diet (Skretting, Stavanger, Norway) in side-by-side 5.3 m^3^ circular fiberglass tanks (approximately 25 kg m^−3^stock density). Rearing temperature was progressively increased to 12 °C in accordance with Norwegian aquaculture industry standards and maintained at 12 °C throughout the experiment. At 3 g size (bulk weighed), fish were graded to obtain homogenous populations with respect to body mass/fork length and to maintain stocking density (35 kg m^−3^). At 25 g size, and 2 weeks prior to swim screening, 600 fish per strain were selected to limit the variance in body mass and fork length to ±3 g and ±1 cm, respectively, and individually tagged with a passive integrated transponder (Jojo Automasjon ÅS, Sola, Norway). Each stock was then reared in five replicate circular tanks (0.1 m^3^, *n* = 120 per strain, 36 kg m^−3^ stock density) until tested for swimming performance and exercise training. Throughout, water exchange and current were set and routinely adjusted to self-clean the tanks in accordance with standard procedures and to provide a nominal water current [0.2–0.3 fork lengths (*FL*) s^−1^]. Specific growth rates were 3.40 and 3.07 for the Bolaks and Lærdal strains, respectively, during this period. Therefore, to minimize the size dichotomy, the faster growing Bolaks strain were screened for swimming performance and exercised 2 months ahead (September 2014) of the Lærdal strain (November 2014).

### Swimming performance and exercise training

The swimming performance and exercise experiments are described in detail elsewhere [30]. In brief, the swimming performance test was performed on groups of 60 fish (fasted for 24 h) per run in Brett-type swim tunnels (29 kg m^−3^ stock density). After 4 h habituation (water current of 0.5 *FL* s^−1^ without tail beats) water velocity was incremented by 5 cm s^−1^ every 10 min until all the fish had reached exhaustion (typically ≤145 cm s^−1^). Fatigued fish were immediately removed via a hatch situated above the back grid and recorded for pit-tag, body mass, fork length, final water speed (*U*_max_) and swimming duration. For each population, the first 20% and last 20% of each group of 60 fish per run to reach fatigue were categorized as inferior and superior swimmers respectively, and transferred to their original rearing tanks for 48 h recovery prior to exercise.

The exercise training experiment lasted for 18 days in the same swim tunnels, each containing 40 inferior and 40 superior swimmers (38 kg m^−3^ stock density). For each population, one swim tunnel was used for aerobic exercise training, which involved maintaining the water velocity at 2 *FL* s^−1^ for the first 7 days, at 2.4 *FL* s^−1^ for next 7 days and at 2.8 *FL* s^−1^ for the last 4 days. The other swim tunnel (water velocity of 0.5 *FL* s^−1^) was used for control fish so that these fish spread themselves along the length of the swim tunnel and only swam occasionally (using slow and small-amplitude tail beats to move forward)*.* Fish were fed a daily ration of 2% biomass through a hatch situated above honeycomb grid at the front of the swim tunnels, which was connected to an automatic belt feeder. After experiments, fish were transferred to their original rearing tanks for 5 days recovery before being sacrificed (decapitation) and sampled for organs.

### Sample preparation and sequencing

Heart ventricles (from 117 animals total, Table 1) were dissected out using a scalpel, blotted dry on tissue paper and immediately snap-frozen in liquid nitrogen for storage at −80 °C. Libraries for RNA-seq were prepared according to Illumina guidelines for the TruSeq Stranded mRNA LT sample preparation kit (TruSeq Stranded mRNA_seq_PE_100bp_FC work sheet, Illumina, San Diego, USA). RNA integrity was assessed using an Agilent 2100 Bioanalyzer with RNA Nano kits (Agilent Technologies, Santa Clara, CA, USA). A total of 8 lanes were run, with 16 fish (libraries) per lane (the final lane was filled with additional samples for another study). Samples with RNA integrity values greater than 8 were accepted for further analysis. The concentration of RNA was determined by Nanodrop A_260_ measurement and 400 ng total RNA was used as input for RNA-seq. The libraries produced were sequenced using 101 cycles for read 1, 7 cycles for the index read and 101 cycles for read 2. Reads were processed using default parameters in Trimmomatic version 0.32 [31] before being aligned to the Atlantic salmon reference genome (3.6 assembly, version GCA_000233375.4, [32]) using Bowtie2 version 2.2.3 [33].

**Table 1:** Experimental factors and states (number of fish in parentheses)

Factor	State 1	State 2
Exercise regime	Control (60)	Trained (57)
Swimming performance	Inferior (58)	Superior (59)
Sex	Male (57)	Female (53)
Source	Lærdal (wild, 57)	Bolaks (domesticated, 60)

### Differential gene expression analysis

The overall model of analysis used was,

$$ \mathrm{Y}\sim \mathrm{Sex}+\mathrm{Source}+\mathrm{Exercise}\_\mathrm{Regime}+\mathrm{Swimming}\_\mathrm{Performance} $$

The analysis was also run on subsets of data (males, females, Lærdal source, Bolaks source, control, trained, inferior swimming performance and superior swimming performance).

Gene coverage data under different states were compared for three experimental factors, exercise regime, swimming performance and sex as outlined in Table 1, using the DESeq2 package [34] in the statistical R programming language [35]. Results were represented as Volcano and Bland-Altman MA scale plots where,

$$ {\log}_2\hbox{-} \mathrm{fold}\  \mathrm{change}\  \mathrm{in}\  \mathrm{expression}\ \left(\mathrm{M}\right)={\log}_2{\mathrm{CD}}_{\mathrm{state}2}\hbox{-} {\log}_2{\mathrm{CD}}_{\mathrm{state}1} $$

and

$$ \mathrm{average}\  \mathrm{expression}\  \mathrm{across}\  \mathrm{states}\ \left(\mathrm{A}\right)=0.5\ast \left({\log}_2{\mathrm{CD}}_{\mathrm{state}2}+{\log}_2{\mathrm{CD}}_{\mathrm{state}1}\right) $$

where CD_state1_ and CD_state2_ were the average coverage depths for each gene under the comparison states 1 and 2 that are described in Table 1. The log_2_-fold change estimate (M) indicated how the gene expression has changed in state 2 relative to state 1. DESeq2 was used to detect and correct low dispersion estimates by modelling the dependence of dispersion on the average expression strength (A) over all the samples. M was estimated as a “shrunken fold change” where,

1. gene-wise dispersion estimates were generated independently for each gene,
2. the expected dispersion value for genes of a given expression strength were estimated, and,
3. the gene-wise dispersion estimates were shrunk towards the values predicted by the expected dispersion curve to obtain final dispersion values using an empirical Bayes approach where the extent of shrinkage depends on the fit of true to expected dispersion and degrees of freedom [34].

This method was applied to reduce bias caused by the strong variance of log-fold change estimates for genes with low average read counts, and has been shown to improve the stability and reproducibility of analysis (compared to maximum-likelihood methods) [34].

Differential expression was tested using a Wald test where the shrunken estimate (M) was divided by its standard error (producing a *z*-statistic that can be compared to a standard normal distribution) [36, 37]. *P*-values were adjusted for multiple testing using a Benjamini and Hochberg procedure [38].

A principle component analysis of the DESeq2 normalised and regularised-logarithmic transformed read abundance data [34] was conducted to visualise the influence of the different variates (swimming performance, exercise and source) on overall patterns of gene expression.

Gene Ontology (GO) terms and gene names were assigned using Blast2GO [39] against the UniProt/TrEMBL database. A gene enrichment analysis was performed by comparing the proportion of differentially expressed genes assigned to each GO annotation for each contrast to the overall assignment of the full set of genes expressed in all states. Enrichment was tested using a 1-tailed chi-square test with Yates’ correction for continuity. The proportion of differentially expressed genes assigned to each GO annotation for each contrast were represented in spider web (radar) plots.

### Detection of putative SNP loci from sequence data

Freebayes software was used to detect variants in the sequence alignments relative to the reference allele [40]. Homozygous reference genotypes were detected using a custom pipeline which identified all positions containing variants and extracted sequence coverage data from the sequence alignment map. The output of this custom pipeline contained information about contig ID, SNP position, reference and alternate allele type, total read depth, read count for the reference and alternative alleles, sum of read qualities for the reference and alternative alleles and sequence quality scores. This data was used to identify likely sequencing errors and to score the genotypes at each SNP position.

An R script was written to discard genotypes with less than 10 X coverage, and putative heterozygotes, where the proportion of counts for either of the alternative alleles was less than 20%. Further filters were applied across the population of fish tested to reject loci where less than 90 or more alleles were detected (such that less than 45 individuals were fully genotyped) and instances where all animals were scored as heterozygotes (all animals shared the same genotype for the locus). Finally, only SNP loci with more than two occurrences of the alternative allele were retained for analysis.

### SNP allele similarity between fish

From a randomly selected 1000 putative SNP loci, 106 (with >60% animals genotyped) were used to compare genetic relatedness by calculating dissimilarity indices using the DARwin software package [41, 42] and to plot a Neighbour Joining Tree using MEGA 6.0 software [43].

### Outlier loci

Separate comparisons were made to identify outlier SNP loci under differential selection between the Lærdal River and Bolaks strain sources. The annotation for each SNP was derived by linking the contig_ID and position fields identifying each SNP to the corresponding annotated contig gene feature range in the reference salmon genome map. Genomic features such as protein coding sequence (CDS), five-prime untranslated region (5’ UTR) and three-prime untranslated region (3’UTR), were captured for each region containing a SNP. Other features such as “exon”, “mRNA” and “gene”, which were not useful annotations in this instance, were removed.

The software BayeScan [44] was used to identify outlier loci under selection by using logistic regression to decompose locus-population FST coefficients into their population component (beta, shared by all loci) and locus-specific component (alpha, shared by all populations). Departure from neutrality is assumed when alpha differs significantly from zero (positive values indicate diversifying selection, negative balancing or purifying selection). The software implements a reversible-jump Markov Chain Monte Carlo algorithm to estimate the posterior probability under these models. The MCMC algorithm was set to use 5000 outputted iterations, thinning interval of 10, 20 pilot runs of 5000 length, burn-in length of 5000, prior odds for the neutral model of 500, lower bound for uniform prior on Fis 0, upper bound 1 and threshold for the recessive genotype as a fraction of maximum band intensity of 0.1.

A Chi-square test for heterogeneity was used to test for allele frequency differences between the inferior and the superior wild Lærdal swimmers.

### Functional interpretation of data

For the functional interpretation of data, differentially expressed genes were considered as those with adjusted *P*-values (*P*_adj_) less than 0.05 and log-fold changed expression (log2-FC) greater than 1.0, and were annotated using KEGG, GO and custom STARS vocabulary [45]. Annotated genes were assigned to multiple functional categories and pathways at the cellular and tissue/systemic levels.

## Results

### RNA-seq and sequence assembly

RNA integrity values were high (>9) for all samples, and an average of 32.5 million reads per paired-end flow cell (ie. 16.25 million reads of each RNA fragment) was achieved (Additional file 1). The RNA sequences were assembled into 58,473 gene loci comprising 364,486 transcripts with an average transcript length of 7957 bases. The counts at each gene loci for the technical replicates was highly correlated (*r* = 0.9994). Average coverage over all samples and loci was 162 ± 4777 (mean ± standard deviation). BLASTX provided 11,911 unique gene name terms.

### Differential gene expression between domesticated (Bolaks) and wild (Lærdal) strains

A total of 2515 genes (11,868 transcripts) were differentially expressed between the hearts sampled from Lærdal and Bolaks salmon (*P*_adj_ < 0.05 and log2-FC > 1, Additional file 2). As much as −11-fold average down- and 10-fold average up-regulation was observed. Based on adjusted *P*-values, the most differential expression occurred in signalling pathways, paracrine regulation genes (growth factors), several metabolic pathways (metabolism of xenobiotics, lipids, steroids and bile) and the immune system. While most functional groups included both up- and down-regulated genes, several immune categories showed a clear trend to lower expression in domesticated Bolaks salmon, especially immunoglobulins, complement system and innate antiviral responses (Table 2), and the number of down-regulated genes in these categories was higher than in wild Lærdal salmon. Table 3 shows examples of differentially expressed immune genes. A tendency for lower expression in Bolaks salmon was also shown by other functional groups involved in protection (stress responses, metabolism of xenobiotics), cell cycle regulation and metabolic pathways, while consistently higher expression was observed for markers of erythrocytes (18 transcripts with mean 1.8-fold differences) (Table 4).

**Table 2:** Average fold differential cardiac gene expression (*P*_adj_ < 0.05) for functional groups affecting immunity between Bolaks and Lærdal parr. Numbers of down-and up-regulated genes in Bolaks relative to Lærdal parr are shown

Category	Down	Up	Fold
Cytokine & receptor	18	10	−1.17
Chemokine & receptors	16	9	−1.25
Eicosanoid	10	5	−1.24
Acute phase	13	2	−2.07
Innate antiviral response	91	2	−1.89
TNF related	19	5	−1.49
Complement	53	3	−2.31
Effector	39	8	−1.61
Antigen presentation	22	1	−1.65
T-cell & lymphocyte	21	8	−1.33
Immunoglobulins	118	2	−2.27

**Table 3:** Examples of differentially expressed immune genes (*P*_adj_ < 0.05) between Bolaks and Lærdal parr

Gene	Fold	Gene	Fold
Serotransferrin-1	−3.54	IL-8	−2.09
Nattectin	−3.53	Arachidonate 12-lipoxygenase, 12S–type	−2.33
C-reactive protein similar, pentraxin	−3.11	Epidermis-type lipoxygenase 3	2.77
MHC class I antigene	−2.06	IFN-induced with tetratricopeptide repeats 5–1	−2.81
HLA class II HC antigen, DP alpha chain	−2.04	Ubiquitin protein ligase E3A	−2.61
C-C chemokine receptor type 3	−4.27	Sacsin	−2.32
CCL4-like chemokine	−2.94	Interferon-induced protein 44	−2.21
C-C motif chemokine 13	2.04	myxovirus resistance 1	−2.05
Chemokine CCL-C11b	2.10	NILT4 leukocyte receptor	−2.95
Complement component C3–3	−3.23	Novel immune-type receptor 3	−2.81
Properdin P factor 3	−3.12	Leukocyte antigen CD37	−3.05
Mannan binding lectin serine proteases	−3.11	POU domain class 2-associating factor 1	−2.54
Complement component C5	−3.07	CD276 antigen	3.58
IL-1 beta	−2.33	CD40 (TNFR superfamily member 5)	−7.42

**Table 4:** Examples of fold differences in gene expression (*P*_adj_ < 0.05) between Bolaks and Lærdal parr for different functional groups

Gene	Fold	Gene	Fold
*Cell cycle, stress responses, xenobiotic metabolism*		Cathepsin L	−3.03
Cyclin-dependent kinase inhibitor 1C	−2.25	MASP2-like serine protease	−3.03
DNA replication licensing factor MCM3	−2.14	Alpha-1-antiproteinase-like protein	−3.46
Cyclin-dependent kinase 4 inhibitor D	−2.07	Alpha-2 macroglobulin-like protein	−3.05
Retinoblastoma binding protein 5	−2.03	7-dehydrocholesterol reductase [7-DHC reductase]	−3.94
Heat shock protein 30	−3.91	Cytochrome P450 2 M1	−3.02
Heat shock protein beta-11	−3.91	Testosterone 17-beta-dehydrogenase 3	−2.78
Heat shock protein Hsp-16.1/Hsp-16.11	−2.81	Vitamin D3 hydroxylase-associated protein	−2.28
Heat shock 70 kDa protein 4	−2.75	*Hormones, blood coagulation*	
Immediate early response 2–2	−2.64	Type II iodothyronine deiodinase	−2.62
GADD45 alpha	−2.39	Natriuretic peptide receptor type B	−2.45
AP1–1	−2.22	Ventricular natriuretic peptide	−2.38
UDP glucuronosyltransferase 2a1–3	−3.97	Plasminogen	−2.95
Aryl hydrocarbon receptor 1 beta-like	−2.85	Coagulation factor IX precursor	−2.88
Cytochrome P450	−2.75	Coagulation factor X precursor	−2.82
Glutathione S-transferase theta-1	−2.40	Coagulation factor V	−2.82
*Lipid and steroid metabolism, proteases and inhibitors*		Coagulation factor X precursor	−2.70
Polyunsaturated fatty acid elongase	−4.34	*Erythrocytes*	
Alpha-tocopherol transfer protein	−2.81	Band 3 anion exchange protein	1.80
polyunsaturated fatty acid elongase elov12	−2.79	Rhag	1.62
Very long-chain acyl-CoA synthetase	−2.73	Erythrocyte membrane band 4.1	1.58
Low-density lipoprotein receptor-related p	−2.73	Carbonic anhydrase, CAH	2.49
Carboxypeptidase B precursor	−3.19	Hemoglobin subunit alpha	1.73
		Hemoglobin subunit beta-1	4.12

### Differential gene expression associated with swimming performance

**Fig. 2:**
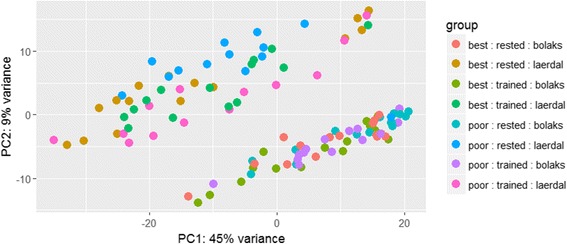
Principle component analysis (PCA) of DESeq2 normalised and transformed (regularised-logarithm) read abundance data. Variables: swimming performance (best/superior or poor/inferior); exercise (rested/control or trained) and; source (Bolaks or Lærdal)

**Fig. 3:**
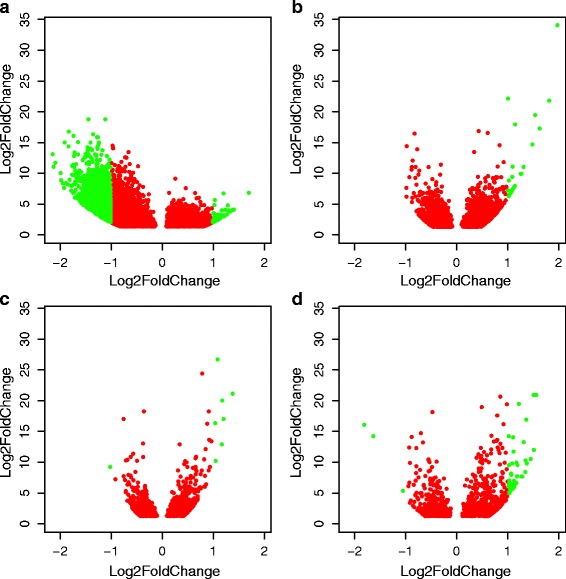
Volcano plots of adjusted *P*-values (*P*_adj_) and log fold change in expression (log_2_FC) of genes in the superior compared to the inferior swimming parr. Separate plots are for wild Lærdal parr under control (**a**) and trained (**b**) exercise conditions, and for domesticated Bolaks parr under control (**c**) and trained (**d**) exercise conditions. Red points show genes with *P*_adj_ < 0.05 while green points show genes with *P*_adj_ < 0.05 and log_2_FC > 1

Under the full analysis model (accounting for sex, source and exercise regime) no genes were differentially expressed in the heart when comparing parr grouped on superior or inferior swimming performance (*P*_adj_ < 0.05 and log_2_FC < 1). Principle component analysis showed that samples clustered with other samples from the same source population with the first component explaining most (45%) of the variance (Fig. 2). The overall pattern of cardiac gene expression with swimming performance depended on exercise regime (trained or control) and source population (Lærdal or Bolaks) (Fig. 3). A larger number of genes were down-regulated (1499 with *P*_adj_ < 0.05 and log_2_FC < 1 compared to 482 with *P*_adj_ < 0.05 and log_2_FC > 1) in superior swimmers among the Lærdal control group (Additional file 3). In contrast, trained Lærdal, control Bolaks and trained Bolaks parr all showed similar patterns of differential expression between swimming performers (Additional files 4, 5 and 6), with more genes showing higher expression (21, 7 and 42 genes respectively with *P*_adj_ < 0.05 and log_2_FC > 1) and very few genes showing lower levels of expression (0, 1 and 3 genes respectively with *P*_adj_ < 0.05 and log_2_FC < 1) in the superior swimmers of trained Lærdal, control Bolaks and trained Bolaks (Fig. 3).

**Fig. 4:**
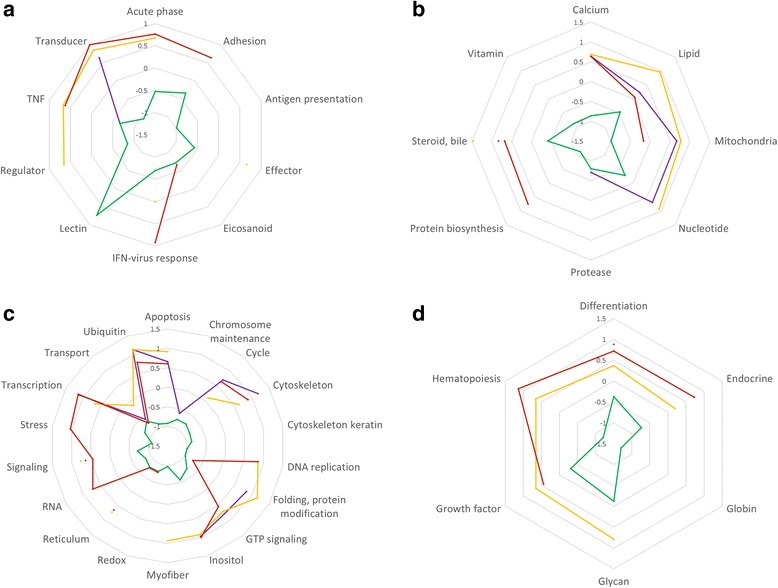
Spiders web plots showing the average differential expression (*P*_adj_ < 0.05) of genes in parr subjected to training versus control exercise regimes. Functional categories are grouped according to immune function (**a**), metabolism (**b**), cell (**c**) and tissue (heart, **d**). Points and joining lines are for wild Lærdal superior performing (green) and wild Lærdal inferior performing (red), domesticated Bolaks superior performing (purple) and domesticated Bolaks inferior performing (yellow) parr. TNF, tumour necrosis factors. IFN, interferon. GTP, guanosine triphosphate

Genes with immune function (acute phase, adhesion, antigen presentation, effector, eicosanoid, signalling, innate antiviral response, regulator and transducer TNF-related) were on average significantly down-regulated in the superior versus inferior swimming performance Lærdal control parr, whereas the opposite expression pattern was generally observed for immune functional categories in Bolaks control, Bolaks trained and Lærdal trained parr (Fig. 4a). Similar differences in expression were generally observed in metabolism, cell and tissue (heart) functional categories (Figs. 4b-d).

**Fig. 5:**
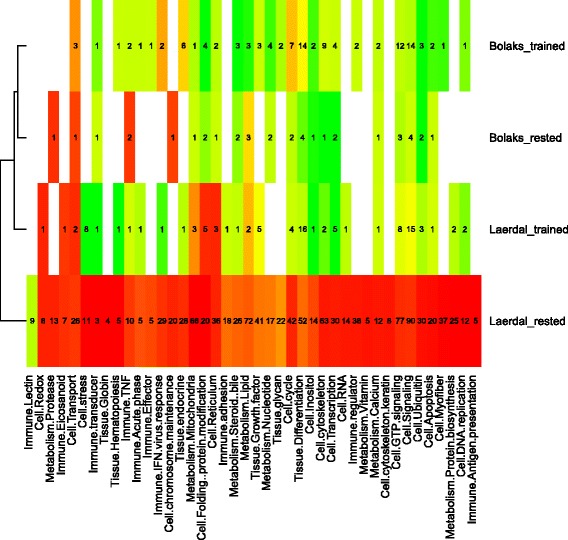
Heat map of differentially expressed (*P*_adj_ < 0.05) functional categories of genes in parr subjected to training versus control exercise regimes. Average log-fold difference in gene expression within each functional category is represented on a colour scale from red (down-expression in the trained parr) to green (up-expression in the trained parr). The heat map is split into domesticated Bolaks and wild Lærdal superior and inferior swimming parr. Numbers of significantly differentially expressed genes in each category are shown

The functional categories showing reduced expression in control Lærdal parr compared to the other states related mostly to cellular stress signalling, immune functions and general metabolism (steroid bile, lipid, nucleotide, vitamin, calcium and protein synthesis) (Fig. 5, Additional file 7). Down-regulated genes of interest in parr with the superior swimming performance among the *L*æ*rdal* control group included transcription factors AP-1 and jun-D, hemoglobin subunit alpha, CEF10, Cox8b (cytochrome c oxidase polypeptide VIII-heart) and Hsp11b (heat shock protein beta-11) (Table 5). These fish also showed a number of up-regulated genes including Immune costimulatory protein, Epithelial cadherin, Cytochrome P450 family 2 subfamily 1 polypeptide 23, T-box Fibronectin, Neuromodulin and Complement C1q-like protein 2 (Table 5).

**Table 5:** Annotated differentially expressed genes (*P*_adj_ < 0.05 & log_2_FC > 1) for the superior versus the inferior swimming performers

Down-regulated	Up-regulated	Down-regulated	Up-regulated
*L*æ*rdal control*		*L*æ*rdal trained*	
*Transcription factor AP-1; Transcription factor jun-D; Hemoglobin subunit alpha; CEF10; Cytochrome c oxidase polypeptide VIII-heart; Heat shock protein beta-11; Acetyl-coenzyme A synthetase 2-like; 14–3-3 protein gamma-2; Putative tropomyosin; AP-3 complex subunit beta-1; Calcineurin B homologous protein 2; Calcium-binding protein p22; Serine/threonine-protein kinase pim-3;* etc.	*NDRG1; Transposase; Fructose-1; Lipolysis-stimulated lipoprotein receptor; Immune costimulatory protein; PiggyBac transposase; Inter-alpha-trypsin inhibitor heavy chain H3; Connective tissue growth factor-like protein; Tetraspanin-1; Epithelial cadherin; Cytochrome P450; Protein cordon-bleu; Polyunsaturated fatty acid elongase elovl5b; T-box transcription factor TBX2b; Fibronectin; Phospholipase A1 member A; Neuromodulin; Complement C1q-like protein 2; Deoxyribonuclease gamma*	*Nil*	*Plasminogen activator inhibitor 1; Cyclic AMP-dependent transcription factor ATF-3; Early growth response 1; Transcription factor AP-1; Tartrate-resistant acid phosphatase type 5; Krueppel-like factor 2; Smtnl protein; DNA damage-inducible transcript 3*
Bolaks *control*		Bolaks *trained*	
*Snail homologue 3*	*Smtnl protein; Tartrate-resistant acid phosphatase type 5; Cyclic AMP-dependent transcription factor ATF-3*	*Snail homologue 3; Alpha-1D adrenoreceptor*	*Serine/threonine-protein kinase 6; Smtnl protein; HMG-CoA reductase; Tartrate-resistant acid phosphatase type 5; F3a protein; Krueppel-like factor 15; Serine/threonine-protein kinase Pim-1; Cysteine dioxygenase; Thromboplastin; Cryptochrome 3*

Among the trained *L*æ*rdal*, control Bolaks and trained Bolaks parr, the snail homologue 3 and alpha 1D adrenergic receptor were the only two annotated genes that were down-regulated (*P*_adj_ < 0.05 and log_2_FC < 1) while several different annotated genes were up-regulated (*P*_adj_ < 0.05 and log_2_FC > 1) (Table 5).

### Comparison of differential gene expression associated with training

**Fig. 6:**
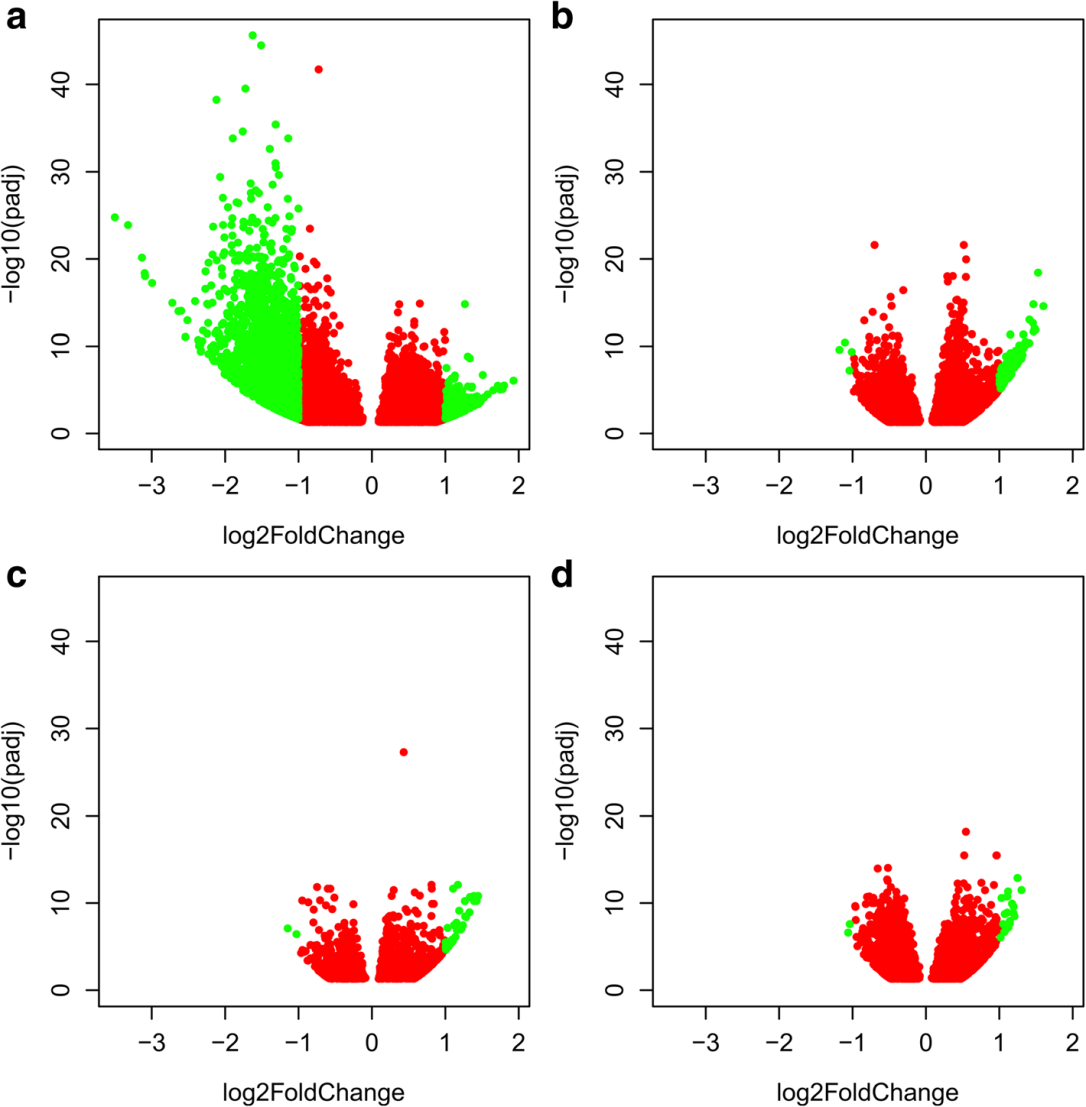
Volcano plots of adjusted *P*-values (*P*_adj_) and log fold change in expression (log_2_FC) of genes in parr subjected to trained compared to control exercise regimes. Separate plots are for wild Lærdal parr with inferior (**a**) and superior (**b**) swimming performance, and for domesticated Bolaks parr with inferior (**c**) and superior (**d**) swimming performance. Red points show genes with *P*_adj_ < 0.05 while green points show genes with *P*_adj_ < 0.05 and log_2_FC > 1

The patterns of differential gene expression observed in trained versus control parr also differed markedly depending on population source and initial level of swimming performance (Fig. 6). Inferior swimming performance Lærdal parr down-regulated large numbers of genes with training (2554 genes with *P*_adj_ < 0.05 and log_2_FC < 1) and up-regulated many genes with training (233, *P*_adj_ < 0.05 and log_2_FC > 1) (Fig. 6a, Additional file 8). In contrast, in the superior swimming performance Lærdal, inferior swimming performance Bolaks and superior swimming performance Bolaks parr (Additional files 9, 10 and 11) there were a few genes that were down-regulated with training (4, 2 and 2 respectively, *P*_adj_ < 0.05 and log_2_FC < 1) and many genes up-regulated with training (103, 42 and 19 respectively, *P*_adj_ < 0.05 and log_2_FC > 1) (Figs. 6b-d respectively). Larger fold-changes in differential expression were observed in Lærdal parr (Figs. 6a-b) than Bolaks parr (Figs. 6c-d), especially in inferior swimming performance Lærdal parr (Fig. 6a).

**Fig. 7:**
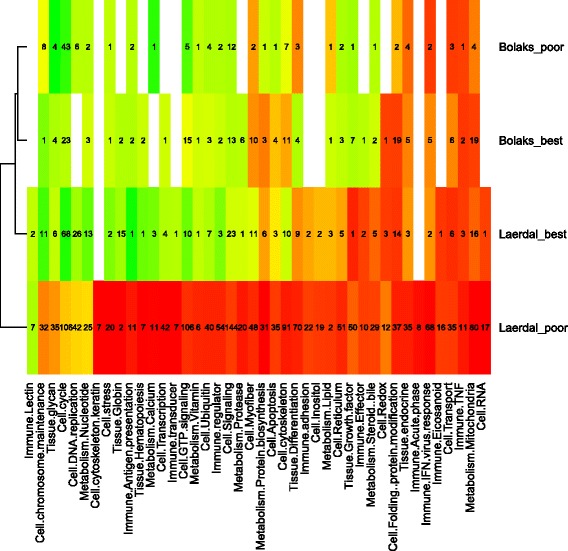
Heat map of differentially expressed (*P*_adj_ < 0.05) functional categories of genes in the superior versus the inferior swimming parr. Average log-fold difference in gene expression within each functional category is represented on a colour scale from red (down-expression in the superior swimming performers) to green (up-expression in the superior swimming performers). The heat map is split into domesticated Bolaks and wild Lærdal trained and rested (control) parr. Numbers of significantly differentially expressed genes in each category are shown

**Fig. 8:**
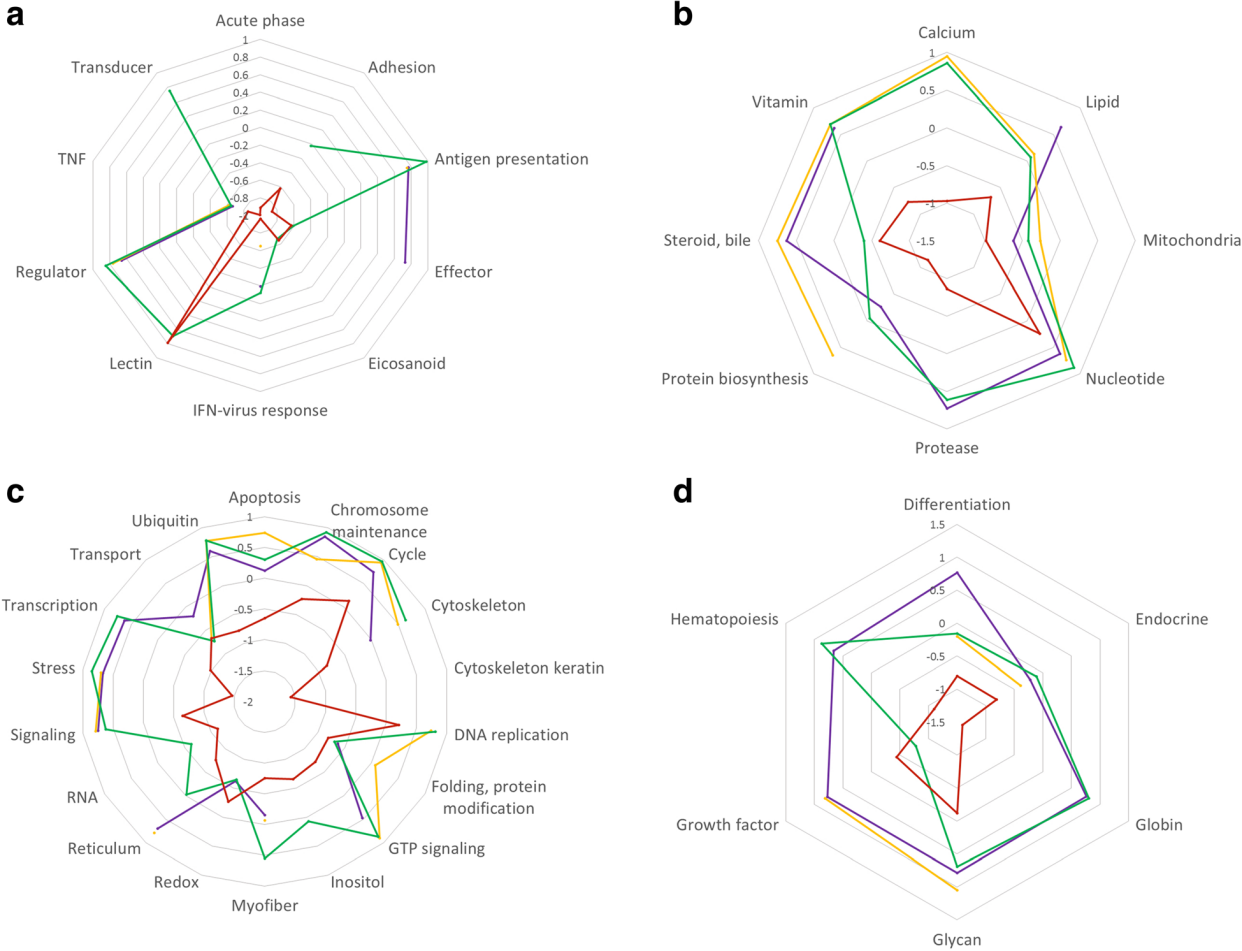
Spiders web plots showing the average differential expression (*P*_adj_ < 0.05) of genes in the superior versus the inferior swimming parr. Functional categories are grouped according to immune function (**a**), metabolism (**b**), cell (**c**) and tissue (heart, **d**). Points and joining lines are for wild Lærdal control (green), wild Lærdal trained (red), domesticated Bolaks control (purple) and domesticated Bolaks trained (yellow) parr. TNF, tumour necrosis factors. IFN, interferon. GTP, guanosine triphosphate

The functional categories of genes that were down-regulated with training in inferior swimming performance Lærdal parr compared to other categories were those associated with cell stress, metabolism and maintenance and immunity (Figs. 7 and 8).

Annotated genes down-regulated with training among the inferior swimming performance Lærdal group included Myosin 1, Early growth response 1, and Beta-actin (Table 6). Several genes were up-regulated among the inferior swimming performance Lærdal (Table 6). The few annotated genes that were down-regulated with training in superior performing Lærdal, inferior performing Bolaks and superior performing Bolaks groups were annotated as Heat shock protein 90-alpha 1 & alpha 4, Agouti related protein-2, and Transferrin receptor protein 1 (Table 6).

**Table 6:** Annotated differentially expressed genes (*P*_adj_ < 0.05 & log_2_FC > 1) for the trained versus the control exercise regimes

Down-regulated	Up-regulated	Down-regulated	Up-regulated
*L*æ*rdal inferior*		*L*æ*rdal superior*	
*Transcription factor AP-1; Transcription factor jun-D; Cyclic AMP-dependent transcription factor ATF-3; CEF-10; Immediate early response gene 2 protein; Serine/threonine-protein kinase pim-3; Myosin 1; Early growth response 1; NADH dehydrogenase 1 beta subcomplex subunit 4; ATPase family AAA domain-containing protein 3; Beta-actin;* etc.	*CD200; DNA replication licensing factor MCM3; NDRG1; Neuromodulin; 11-beta-hydroxylase; Reverse transcriptase-like protein; Inter-alpha-trypsin inhibitor heavy chain H3; Apelin receptor A; C-FLIP AMPA glutamate; T-box transcription factor TBX2b; N-methyl-D-aspartate receptor subunit; FAM131B; Deoxyribonuclease gamma; Voltage-gated calcium channel subunit Cav2.2 variant II; MAGUK p55 subfamily member 2; Neurexin-1-alpha; G1/S-specific cyclin-E2; Carboxypeptidase A6;*	*Heat shock protein 90-alpha 1 & alpha4*	*TC1-like transposase; CD200; Targeting protein for Xklp2; Ubiquitin-conjugating enzyme E2 C; Smtnl protein; Kinesin family member C1; G2/mitotic-specific cyclin-B1; Cell division control protein 2 homolog; Anln-like protein; Regulator of cytokinesis 1; Securin; Baculoviral IAP repeat-containing protein 5; Cytoskeleton-associated protein 5; Epithelial cell transforming sequence 2 oncogene; Deoxycytidine kinase 2; Plasminogen activator inhibitor 1; Borealin; Spindle and kinetochore-associated protein 1; Mki67 protein; Lymphokine-activated killer T-cell-originated protein kinase homolog; SHC SH2-domain binding protein 1; Rac GTPase-activating protein 1; Citron Rho-interacting kinase; Aggrecan core protein; Forkhead box M1; Regulator of cytokinesis 1; DNA repair protein RAD51 homolog A; Hemoglobin subunit alpha; Kinetochore protein Spc25; Inner centromere protein B*
Bolaks *inferior*		Bolaks *superior*	
*Agouti related protein-2*	*G2/mitotic-specific cyclin-B1; Ubiquitin-conjugating enzyme E2 C; Cell division control protein 2 homolog; Anln-like protein; Mitotic kinesin-like protein 1; Kinesin family member 23; Baculoviral IAP repeat-containing protein 5; TC1-like transposase; Securin; SHC SH2-domain binding protein 1; Cell division cycle protein 20 homolog; Kinesin family member C1; Lymphokine-activated killer T-cell-originated protein kinase homolog; Cytoskeleton-associated protein 5; Targeting protein for Xklp2; Bardet-Biedl syndrome 7 protein; Cyclin-A2; Myosin 1; Inner centromere protein B*	*Transferrin receptor protein 1; Transposase*	*BCL2; Ubiquitin-conjugating enzyme E2 C; CDP-diacylglycerol--serine O-phosphatidyltransferase; G2/mitotic-specific cyclin-B1; Collagenase 3; Securin; Cell division control protein 2 homolog; 5′-nucleotidase domain containing 2*

### Detection of SNP loci from sequence data

Out of the 3,059,322 potential sequence variants identified by FreeBayes, 244,466 putative SNP loci remained after filtering criteria and were used for sequence variant analysis across all 192 animals. The mean and standard deviation of pairwise genomic dissimilarity values were similar when comparing animals within the control and trained experimental groups, such that the genetic background of the two experimental groups was homogeneous and therefore unlikely to bias the experimental results.

Higher average genomic similarity was detected for pairwise comparisons of animals within Bolaks or within Lærdal source populations than for pairwise comparisons between the two different sources (dissimilarities of 0.31 ± 0.09 and 0.38 ± 0.05 mean ± standard deviation for comparisons within source and comparisons between source respectively). No difference in pairwise genomic similarity was detected within Bolaks and Lærdal sources (dissimilarities of 0.32 ± 0.08 and 0.30 ± 0.09 mean ± standard deviation respectively).

For the analysis of SNP allele similarity, Lærdal River sourced fish were assigned to a separate clade to that of Bolaks selectively bred fish. Several major groupings within the Bolaks and Lærdal sourced fish were observed.

### SNPs under diversifying selection in Bolaks and Lærdal populations

One hundred and thirty-seven SNP loci were identified as putative outliers (all showing evidence of diversifying selection, *q*-value <0.001, Additional file 12). Of the genes located less than 100,000 bases up or down stream of the SNP outliers, there was a clear trend towards changes in metabolism (especially lipid, glycan and amino acid), intracellular trafficking and maintenance of RNA and chromosome (Table 7 and Additional file 13). Very few outlier genes were classified with functions related to signal transduction, immunity, stress, differentiation and development.

**Table 7:** Functional annotations of cds 1000 bp up or down stream from SNP loci identified as outliers

Function	Number of genes
amino acids metabolism	2
chromosome	4
cytoskeleton	2
ECM collagen	1
energy metabolism	1
glycan	5
growth factor	1
immune	3
lipid metabolism	6
metabolism	1
neural	1
protein biosynthesis	1
protein folding	1
RNA processing	4
signaling	2
sugar metabolism	1
trafficking	4
ubiquitin	3
unknown	14

Many of the SNPs that showed >50% allele frequency differences between Bolaks and Lærdal populations (1125 out of 1806 gene ID’s, or 62%) occurred in genes that showed differential gene expression between these two populations (out of 21,476 gene ID’s in the differentially expressed gene table). One hundred and one of the genes showing >50% allele frequency differences between Bolaks and Lærdal populations were also found to be differentially expressed for swimming performance (5% of the 1699 differentially expressed for swimming performance). Five hundred and eighty-three of the genes that showed >50% allele frequency differences between Bolaks and Lærdal populations were also differentially expressed with training (32% of the 10,498 differentially expressed with regard to exercise regime).

### SNP allele frequency differences between the inferior and superior swimming performers in Lærdal

Significant allele frequency differences for several SNP loci were found between the inferior and superior swimming performers among wild Lærdal parr (*P* < 0.05, Table 8). The genes located 100,000 bases up or down stream of these SNPs were annotated as C-C chemokine receptor type 9; Creatine kinase, mitochondrial 1, 1B and 2; Cytochrome P450 CYP2X20; Deoxyribonuclease gamma; Eukaryotic translation initiation factor 4E–binding protein 2; Fast myotomal muscle actin 2; Inorganic pyrophosphatase; Mammal-like melanopsin 2; ORF2-encoded protein; Serine/threonine-protein kinase PRP4 homolog; and Skeletal muscle actin.

**Table 8:** Significant SNP allele frequency differences (*P* < 0.05) detected between the inferior and superior performing parr derived from wild Lærdal stock. All SNPs annotated at these positions occurred in miscellaneous or messenger RNA (non-coding)

Contig (SNP position)	Annotation at position	*P*-value	Inferior		Superior	
			Allele 1	Allele 2	Allele 1	Allele 2
jcf1000653657_0–0 (164943)	serine/threonine-protein kinase PRP4 homolog	0.03415	0	28	8	22
jcf1000919488_0–0 (1135350)	actin, alpha cardiac-like	0.035823	7	5	0	10
jcf1000879147_0–0 (2585651)	40S ribosomal protein S24	0.035935	20	12	23	1
jcf1000492378_0–0 (25572)	–	0.037224	0	16	1	1
jcf1001039889_0–0 (991799)	–	0.041517	8	12	0	16
ccf1000000646_0–0 (114260)	–	0.048667	2	2	0	14
ccf1000000401_0–0 (273699)	–	0.049832	1	19	10	14

## Discussion

### Domesticated salmon show lower expression of immune-related genes

Large numbers of genes with putative immune function were found to have significantly lower expression levels in domesticated (Bolaks) parr than in wild (Lærdal) parr. These differences in gene expression might be influenced by the genetic differences that have accumulated between the domesticated and wild stock, but could also be influenced by what is known as trained immunity [46] for which epigenetic programming leads to sustained changes in the expression of genes influencing the innate immune system (innate immune memory). In either case, the aquaculture environment, or the process of domestication, seems to have reduced the phenotypic plasticity and lowered the immune expression in the Bolaks stock.

If the reduced plasticity and reduced immune gene expression in domesticated stock is the result of genetic differences we might expect to detect SNPs associated with immunity under diversifying selection. Of genes mapping in close proximity to the 137 SNP loci detected to be putatively under diversifying selection in the Bolaks and Lærdal populations, few affected immune function (3), signal transduction (2), stress, differentiation and development (Additional file 3). This suggests that differential selection for immune function, and the accumulation of differences in immune genetics between the two populations, are relatively small. However, only animals from a single wild salmon river population were sequenced in this study and the SNPs that mark loci under divergent selection might occur in the actual genes that are under selection or might be in tight linkage with such genes (ie. closely linked immune genes to these SNPs might not have been detected). Most of the SNPs putatively under divergent selection between Bolaks and Lærdal populations occurred in or mapped near to genes that showed differential gene expression between these two populations, however this is not surprising as a large number of genes were significantly differentially expressed (more than 50% of genes were differentially expressed with *P*_adj_ < 0.01). Consistently higher expression was observed for markers of erythrocytes in the heart of Bolaks salmon (18 transcripts with mean 1.8-fold differences) (Table 4) indicating that either blood circulation and/or erythropoiesis was enhanced in these parr or that the overall conformation of the heart tissue (eg. density) differed between the Bolaks and Lærdal parr. The number of crosses used to generate the 57 Lærdal and 60 Bolaks fish that were compared for this analysis was small, and this dataset is therefore unusual compared to most population genetic studies which collect samples from wild fish living in different study areas (which in most instances would share a more distant common ancestry). Therefore, there is a chance that the outlier loci identified in our study do not exist as outliers in the broader Lærdal and Bolaks populations, and further work is needed to verify these results.

### Swimming performance and gene expression

Swimming performance is likely to have been under intense selection in Lærdal fish. Parr must use intense bursts of swimming to avoid predators in Sognefjord and to quickly find their way to feeding grounds in the sea. Tagged pre-smolt from this river system, the mouth of which is located 144 km along Sognefjord from Norway’s outer coastline, are known to initiate migration earlier, and to move at a faster pace along the main migratory routes, than stocks from rivers that start migration in relatively shorter fjords [21]. Low/downregulated expression of genes involved in general metabolism, stress and immune response was associated with better swimming performance among Lærdal parr that were not exercised (control exercise regime, Figs. 4 and 5). Better performing parr enter what amounts to a kind of “energy conservation mode” after gentle swimming in a continuous 0.5 FL-s current over 18 days (control exercise regime) and are likely better able to sustain the bursts of swimming needed to avoid predators and reach the feeding grounds in the sea. The genes with the most down-regulated expression in better swimming performance parr from Lærdal after the control exercise regime were: haemoglobin subunit alpha transporting oxygen [47]; CEF10 which is related to connective tissue growth factor involved in cell adhesion, migration, proliferation and angiogenesis [48]; cytochrome c oxidase involved in the process of synthesising ATP [49]; and heat shock protein beta 11 (Table 5).

There was also little overall difference in the expression patterns of trained and inactive Bolaks (Fig. 3). Genes that were commonly more highly expressed in parr with the superior swimming performance were: Smtnl protein which is involved in regulating the contraction and relaxation of skeletal and smooth muscle fibres and mediates vascular adaptation to exercise [50]; Tartrate-resistant acid phosphatase type 5 which has been thought to play a role in osteoclast migration [51], generation of reactive oxygen species [52], iron transport [53] and cell growth and differentiation [54]; and, Krueppel-like factor 2 which regulates T-cell trafficking [55] (Table 5). One of the two genes with lower expression associated with superior swimming performance in the trained Bolaks parr was the alpha 1D adrenergic receptor which is known to play a role in regulating the myocardial contractile performance of exercising dogs [56].

### Differential gene expression with exercise

Training is known to dramatically modify the physiology of vertebrates, but the same training regime could have quite different individual effects depending on physical and genetic conformation. Large numbers of genes with functions involved in stress, immune response and general cell metabolism were down-regulated with training over 18 days among the inferior swimming performing Lærdal group, suggesting that the physiology of these parr is substantially modified by training. The differentially expressed genes are more-or-less the same (for example, Hemoglobin subunit alpha, CEF10 and Heat shock protein beta-11), and affected in a similar way, to those displaying differential expression between inferior/superior swimming performers among resting Lærdal parr. This is probably because the inferior swimming performing Lærdal parr react the strongest to training, and in a way that their physiology and gene expression becomes more like the superior performing Lærdal parr. The lowered expression of genes affecting immune response in inferior performing Laerdal parr with hard exercise training could affect the ability of these parr to fight infectious challenges. With this change the immunological state of these Lærdal parr becomes closer to that found in domesticated Bolaks parr, which were also found to have relatively low-level immune gene expression. Castro et al. [13] also found, although not significant, that training had a stronger effect on poor swimmers than good swimmers. As discussed above, the response of the inferior swimming Lærdal parr after 18 days of training is likely toward greater energy efficiency.

Training also has a different effect on the expression of genes in Lærdal and Bolaks sourced parr. Among the Lærdal parr with inferior swimming performance, training resulted in a marked down-regulation of a large number of genes involved in immune function, metabolism, cell function and tissue function (Fig. 8a-d) and up-regulation of some genes involved in cell DNA replication and redox, nucleotide metabolism and immune lectin function (Fig. 6). Overall less fold changes in expression were observed in the superior performing wild parr compared to wild parr with poor swimming performance (Fig. 6a,b). Intriguingly, very little difference was detected between the gene expression changes with training of the inferior and superior swimming Bolaks parr (Fig. 6c and b), and the genes, function, direction and extent of differential expression detected in these parr was similar to that detected in the superior swimming performance Lærdal parr. Less genetic plasticity in response to training was detected among the Bolaks parr than among the Lærdal parr as demonstrated by fewer differentially expressed genes and smaller log-fold changes in expression.

A similar set of genes were down-regulated when comparing training versus control expression in the inferior swimming performance Lærdal parr (Table 6) as was found to be down-regulated in parr with comparison of the superior versus the inferior swimming performance from Lærdal after 18 days of gentle control exercise (Table 5). These genes included Early growth response 1 whose expression in the spinal chord of rats has also been found to be affected by training [57]. In the superior swimming performance parr from Lærdal and Bolaks, and the poorest swimming performance parr from Bolaks, training resulted in reduced expression of Heat shock protein 90-alpha 1 & alpha 4 which are known to be stress inducible molecular chaperones [58], Transferrin receptor protein 1 which is involved with the cellular uptake of iron and development of erythrocytes [59], and Agouti related protein-2 which is only found in teleosts and affects energy homeostasis and pigmentation [60]. Common genes that were more highly expressed under these states included a number of genes affecting mitotic cell division: Ubiquitin-conjugating enzyme E2 C which is known to be highly expressed in the brain and heart [61]; Cell division control protein 2 homolog and G2/mitotic-specific cyclin-B1 which when expressed together induce proliferation of cardiomyocytes [62]; Securin [63, 64]; Targeting protein for Xklp2, Kinesin family member C1 and Cytoskeleton-associated protein 5 which are required for assembly of mitotic spindles [65–67]; Baculoviral IAP repeat-containing protein 5 which promotes cell proliferation and prevents apoptosis [68]; Lymphokine-activated killer T-cell-originated protein kinase homolog which is active in mitosis and involved in phosphorylation of kinases [69]; SHC SH2-domain binding protein 1 which has a role in signalling pathways for cell proliferation growth and differentiation [70]. Other genes of interest that were more highly expressed after training included Haemoglobin subunit alpha [47] and Bardet-Biedl syndrome 7 protein which affects early onset obesity causing hypertension and congenital heart disease [71].

### Comparison of phenotypic plasticity

Less overall plasticity in differential cardiac gene expression with swimming performance and exercise was observed in the domesticated Bolaks versus wild Lærdal parr (Fig. 3). This result fits with our physiological comparison (metabolic enzyme analysis in swimming muscle, respiratory capacity, aerobic scope and oxygen consumption, saturation and levels) which showed that training greatly benefits the wild fish, but produced little benefit for the domesticated fish [30]. Through more than 10 generations of selection in the Bolaks breeding program, there has been no direct selection for enhanced swimming performance. Bolaks stock have been selectively bred in captivity for improved growth rate, condition factor, late maturation and appearance for more than 10 generations [72]. Bolaks fish are derived from two main rivers, the Vosso which leads into a complex fjord system in Hordaland district, and the Årøy which is a short but powerful River that, like the Lærdal River system, drains into Sognefjord more than 100 km from the open sea. Either the source stocks contributing to the Bolaks strain had less phenotypic plasticity or Bolaks parr have lost some of the phenotypic plasticity that was originally present in the wild source stocks during the process of domestication and or genetic drift. The largest gene expression changes with training were detected among the inferior swimmers of the Lærdal parr, which contributed the most to the higher plasticity observed among Lærdal parr.

The wild Lærdal and domesticated Bolaks salmon showed profound differences in gene expression profiling, while similar overall levels of genetic variability were found within the Bolaks and Lærdal populations (similar pairwise genomic similarity at SNP loci). The results suggest that intense selective breeding over several generations, and/or intense adaptive pressures in wild Lærdal river salmon, have affected the expression of multiple functional groups of genes involved in diverse processes at cellular and systemic levels in the heart. Epigenetic effects transmitted from parents to offspring, and random genetic drift, might also account for some of the differences in gene expression observed.

### Genetic variation associated with swimming performance

The genes mapping close to SNPs associated with swimming performance in the wild Lærdal parr included: C-C chemokine receptor type 9 which facilitates cellular signal transduction [73]; Creatine kinase which acts as an energy reservoir for buffering and regeneration of ATP [74]; Cytochrome P450 CYP2X20 which catalyses oxidative reactions and is down-regulated with bacterial infection in catfish [75]; Deoxyribonuclease gamma; Eukaryotic translation initiation factor 4E–binding protein 2 which is involved in synaptic plasticity and protects against pressure overload induced heart failure [76]; Fast myotomal muscle actin 2 and Skeletal muscle actin which code for contractile filaments in the muscle fibre [77]. Some of these genes were also found to have higher expression in the superior swimming performers (Cytochrome P450 and Deoxyribonuclease gamma among the wild Lærdal control group) and similar types of genes were differentially expressed with swimming performance among the other groups (eg. Serine/threonine-protein kinases 6 and Pim-1 oncogene which had higher expression for the superior performers among domesticated trained parr) (Table 5). The precise effects of these SNP variants needs to be investigated further, but such SNPs could be tightly linked to causative mutations affecting the function or expression of these genes, rather than being the actual causative mutations affecting function or expression themselves. None-the-less, further research is needed to verify if the SNPs identified could be useful markers of swimming performance. If there is a genetic component affecting heart shape, or other cardiovascular fitness traits, selective breeding could be used to make genetic improvement so that fish are more robust to stressful situations such as grading, transportation and disease infection.

## Conclusions

Cardiovascular health and performance are important factors affecting the sustainability of Atlantic salmon aquaculture. In this study we used RNA-seq to investigate changes in cardiac gene expression associated with aerobic exercise, swimming performance and genetic origin. The expression of genes with immune related function was found to be suppressed in domesticated Bolaks parr compared to wild Lærdal fish sampled, however no evidence of differential selection at immune related loci was detected. In addition, domesticated parr had less phenotypic plasticity in response to training, and inferior performing Lærdal fish had relatively higher expression of immune genes after control exercise than after hard training. The direction, extent and types of immune genes responding to training were all very different for Lærdal parr with inferior swimming performance. Lower immune gene expression could have negative or positive effects on immune function, depending on the types and combinations of genes whose expression is affected. The extent of the immune response also depends on the nature of the training regime applied to the fish. Continuous regimes have been found to have beneficial effects, whereas stop-start regimes (applied for the same overall swimming miles as continuous regimes) can have detrimental effects [13]. Phenotypic plasticity and immune response to stress are important factors influencing the everyday protection of the fish. More research into the relationship between exercise, cardiovascular gene expression and immune function is needed so that exercise regimes can be tailored in a way that benefits survival and performance in the aquaculture environment.

## Additional files

Additional file 1: Sample and lane statistics from Illumina GAII sequencing. (XLSX 42 kb)

Additional file 2: Differential gene expression detected between Bolaks and Lærdal parr heart. (XLSX 2480 kb)

Additional file 3: Differential gene expression detected for swimming performance among the heart from Lærdal control parr. (ZIP 2534 kb)

Additional file 4: Differential gene expression detected for swimming performance among the heart from Lærdal trained parr. (ZIP 1782 kb)

Additional file 5: Differential gene expression detected for swimming performance among the heart from Bolaks control parr. (ZIP 840 kb)

Additional file 6: Differential gene expression detected for swimming performance among the heart from Bolaks trained parr. (ZIP 613 kb)

Additional file 7: Enrichment analysis for genes differentially expressed for swimming performance in parr. (XLSX 29 kb)

Additional file 8: Differential gene expression detected for exercise regime among the heart from Lærdal inferior swimming parr. (ZIP 4464 kb)

Additional file 9: Differential gene expression detected for exercise regime among the heart from Lærdal superior swimming parr. (ZIP 3092 kb)

Additional file 10: Differential gene expression detected for exercise regime among the heart from Bolaks inferior swimming parr. (ZIP 1115 kb)

Additional file 11: Differential gene expression detected for exercise regime among the heart from Bolaks superior swimming parr. (ZIP 2944 kb)

Additional file 12: Outlier SNP loci showing evidence of diversifying selection (*q*-value <0.001). (XLSX 21 kb)

Additional file 13: Functional annotation of outlier SNP loci showing evidence of diversifying selection (*q*-value <0.001). (XLSX 16 kb)

## Abbreviations

A: average expression
CD: sequencing coverage depth
FL: fork length
GO: gene ontology
M: log-fold change in expression
mRNA: messenger ribonucleic acid
NOK: Norwegian kroner
P_adj_: adjusted *P*-value
RNA: ribonucleic acid
SNP: single nucleotide polymorphism
U_max_: final water speed
